# Dependence of Protein Immobilization and Photocurrent Generation in PSI–FTO Electrodes on the Electrodeposition Parameters

**DOI:** 10.3390/ijms25189772

**Published:** 2024-09-10

**Authors:** Theresa Kehler, Sebastian Szewczyk, Krzysztof Gibasiewicz

**Affiliations:** Faculty of Physics, Adam Mickiewicz University, ul. Uniwersytetu Poznańskiego 2, 61-614 Poznań, Poland; thekeh@st.amu.edu.pl (T.K.); sebastian.szewczyk@amu.edu.pl (S.S.)

**Keywords:** photosystem I, electron transfer, photocurrent, electrodeposition, biophotoelectrode, photoelectrochemical cell

## Abstract

This study investigates the immobilization of cyanobacterial photosystem I (PSI) from *Synechocystis* sp. PCC 6803 onto fluorine-doped tin oxide (FTO) conducting glass plates to create photoelectrodes for biohybrid solar cells. The fabrication of these PSI–FTO photoelectrodes is based on two immobilization processes: rapid electrodeposition driven by an external electric field and slower adsorption during solvent evaporation, both influenced by gravitational sedimentation. Deposition and performance of photoelectrodes was investigated by UV–Vis absorption spectroscopy and photocurrent measurements. We investigated the efficiency of PSI immobilization under varying conditions, including solution pH, applied electric field intensity and duration, and electrode polarization, with the goals to control (1) the direction of migration and (2) the orientation of the PSI particles on the substrate surface. Variation in the pH value of the PSI solution alters the surface charge distribution, affecting the net charge and the electric dipole moment of these proteins. Results showed PSI migration to the positively charged electrode at pH 6, 7, and 8, and to the negatively charged electrode at pH 4.4 and 5, suggesting an isoelectric point of PSI between 5 and 6. At acidic pH, the electrophoretic migration was largely hindered by protein aggregation. Notably, photocurrent generation was consistently cathodic and correlated with PSI layer thickness, and no conclusions can be drawn on the orientation of the immobilized proteins. Overall, these findings suggest mediated electron transfer from FTO to PSI by the used electrolyte containing 10 mM sodium ascorbate and 200 μM dichlorophenolindophenol.

## 1. Introduction

**Figure 1:**
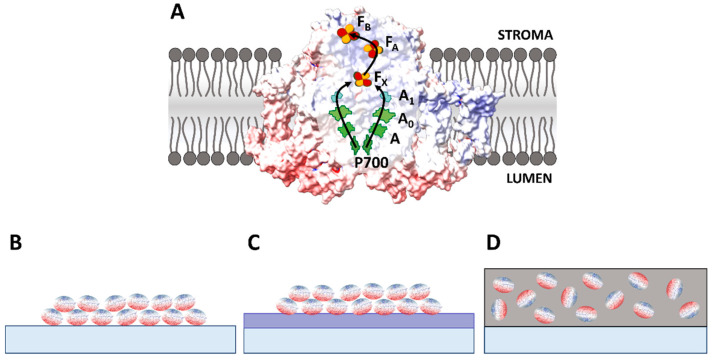
Photosystem I (PSI) in natural (**A**) and exemplary artificial (**B**–**D**) environments. (**A**) PSI embedded in lipid membrane. Native PSI electron transfer cofactors (ETCs) form the reaction center (RC) and are labeled: P700—dimeric chlorophyll *a* (primary electron donor), A/A_0_—monomeric chlorophylls, A_1_—phylloquinone, F_X_/F_A_/F_B_—iron–sulfur clusters, with F_B_ being the terminal electron acceptor. In addition to the indicated ETCs, PSI binds ~90 antenna chlorophyll *a* molecules and 22 β-carotene molecules (not shown for clarity) which harvest the light energy and deliver it to the RC, where electron transfer (ET) is initiated. (**B**) PSI particles deposited directly on a conductive substrate in a quasi-uniformly oriented way. (**C**) PSI particles deposited on a conductive substrate via linker molecules (represented by interfacing rectangle) in a quasi-uniformly oriented way. (**D**) Randomly oriented PSI particles embedded in a matrix coating the conductive substrate. (**A**–**D**) Red and blue colors of PSI surface represent negative and positive surface charges at pH 7, respectively, as concluded from studies on PSI particles isolated from their natural environment (see the main text). (**B**–**D**) White belt around PSI particles is hydrophobic and may bind detergent molecules.

Photosystem I (PSI) is a photosynthetic pigment–protein complex which absorbs light and converts its energy into electric energy of charge-separated states where electrons reach a distance of up to ~6 nm from the positive charge [1,2]. Therefore, it is often tested in different biophotovoltaic systems where it serves as an essential part in generating photocurrent [3,4,5,6,7,8,9]. In intact biological systems, PSI shows exceptionally high efficiency of absorbed photon-to-photoelectron conversion of ~99%, and a significant voltage of ~1 V in its fully charge-separated state [1,2]. However, there are many technological obstacles to utilizing PSI particles in artificial devices. These include difficulties in (1) preserving the natural activity of the protein within the artificial systems [10,11,12,13] and (2) effective coupling of the two opposite sides of the protein (donor or luminal side and acceptor or stromal side, Figure 1A) with artificial external electron donors and acceptors and/or electrodes. As a result, the reported efficiency of conversion of absorbed photons to photoelectrons in such devices rarely exceeds a few percent [14,15]. Yet we believe, that the technological obstacles limiting the potential of PSI protein in large-scale biophotovoltaics are surmountable.

PSI proteins occur naturally in lipid membranes of all plants, algae and some bacteria (Figure 1A). The protein’s electron donor and acceptor sides are hydrophilic. They protrude from the opposite sides of the membrane towards two different aqueous solutions (lumen and stroma) and interact very efficiently with natural electron transfer mediators freely diffusing in these solutions [16,17,18,19,20,21,22,23,24], while the central hydrophobic part interacts with hydrocarbon chains of the membrane lipids. To isolate intact PSI complexes from the membrane, an aqueous solution of mild detergent must be used. The isolated proteins are dispersed in an aqueous solution with their hydrophobic part coated with detergent molecules. In order to facilitate the stable deposition of PSI proteins from this solution onto a substrate, while avoiding aggregation, the detergent concentration in the solution may be reduced through dialysis [13].

Figure 1B–D illustrate a few typical architectures of PSI films on a conductive substrate tested in different biophotovoltaic systems. In the simplest systems, monolayers [25,26] or, more often, multilayers of PSI particles [13,27] are deposited directly on the conductive substrate (Figure 1B). Often, an interfacing layer is introduced between PSI film and the substrate with the intention to enhance binding of PSI and/or properly orient the proteins (Figure 1C; [3,28,29,30]). Figure 1D presents the third architecture in which PSI is embedded in an artificial matrix, e.g., in conductive polymers [31,32], and the mixture of these two forms a film on a substrate.

After charge separation, illuminated PSI protein deposited on the conductive substrate may either collect the electrons from it (by its donor side, see Figure 1A) or (in the opposite geometry) inject electrons into it (by its acceptor side), giving rise to the cathodic or anodic photocurrent, respectively. For that reason, mixed (stromal and luminal) orientation of PSI particles on the substrate is expected to result in a low net photocurrent due to cancellation of the contributions from different orientations. Consequently, it is often claimed that the optimal (for maximal photocurrent generation) orientation of the proteins is uniform with either their luminal (see Figure 1B,C) or stromal side facing the surface of the conductive substrate, but empirical verification of such uniformity of orientation is difficult. One could expect that in the case of the uniform orientation of PSI presented in Figure 1B,C, with the donor side of PSI facing the conductive surface, the cathodic current should be observed. However, many experiments have demonstrated that independently of the expected orientation of the proteins, the observed photocurrent was cathodic under conditions that were non-denaturating for the proteins and with moderate potentials applied to the working electrode (WE) composed of PSI and the conductive substrate [3,15,25,27,29,30,33,34,35]. On the other hand, the studies reporting anodic photocurrent from PSI-based electrodes were conducted either under conditions potentially denaturing proteins [10,11,36], e.g., by using acetonitrile solvent, likely leading to extraction of chlorophylls and direct injection of electrons from these pigments to the conductive substrate, or by application of very high potentials to PSI-based working electrodes [28]. It was convincingly suggested that under high potential applied to the WE, the anodic photocurrent originates not from electron injection from F_B_^−^ to the substrate but rather from electrons injected by excited antenna chlorophylls which possess a very low redox midpoint potential [13,37].

**Figure 2:**
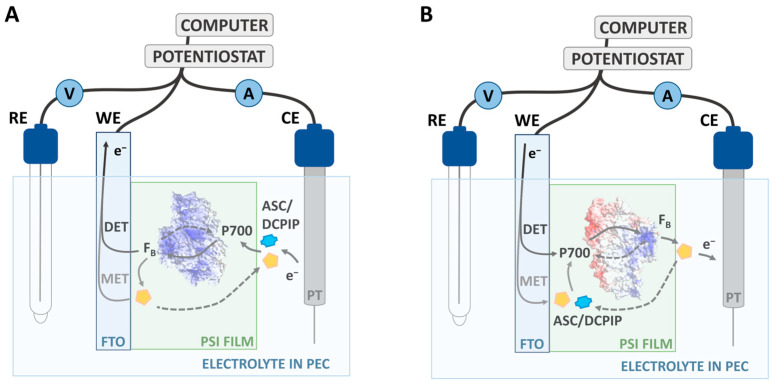
Illustration of direct and mediated ET (DET and MET, respectively) in the photoelectrochemical three-electrode cell based on a PSI–FTO photoelectrode. (**A**) Orientation of the PSI particle with dominating positive surface charge (represented by blue color), with the acceptor side of PSI facing the substrate, leading to anodic photocurrent; such an orientation is assumed at low pH. (**B**) Orientation of PSI particle with a more balanced positive surface charge (on the acceptor side) and negative surface charge (colored in red, on the donor side) with the donor side of PSI facing the substrate, leading to cathodic photocurrent; such an orientation is assumed for neutral or basic pH environments; the more basic the pH, the less positive and more negative surface charges are expected—see text for details. ASC—ascorbate (represented by yellow pentagon); P700—primary electron donor in PSI; F_B_—final electron acceptor in PSI; DET and MET—direct and mediated electron transfer, respectively, between PSI and the substrate of WE. Solid arrows indicate forward ET and dashed arrows indicate backward ET. WE, RE, and CE—working, reference, and counter electrode, respectively.

The above considerations essentially concern PSI proteins that interact directly with the conductive substrate—either as a single monolayer or as the monolayer that is closest to the substrate in a multilayer system (refer to Figure 1B). Such immediate contact opens the possibility of direct electron transfer (DET) between the conductive substrate and the protein. As depicted in Figure 2, the orientation of the PSI particle is essential in DET. However, in the presence of mediators, DET may coexist or be replaced by the mediated electron transfer (MET). In the MET mechanism, the orientation of PSI particles does not determine the direction of the photocurrent. In Figure 2, ascorbate and DCPIP represent the mediators and, for simplicity, only one MET direction is illustrated for each orientation. In a multilayer system, only the first PSI layer directly facing the substrate can generate photocurrent via both DET and MET, whereas all other layers contribute to the overall photocurrent only via MET.

According to these notions, by controlling the orientation of PSI on the substrate, one may influence the direction of photocurrent (cathodic vs. anodic). There are different strategies for exerting this control [25,29,36]. One of them is to exploit the net electric dipole moment of PSI leading to its orientation in an external electric field [13,29,38,39]. One mechanism to enhance this control is by tuning the total surface charge and therefore the net electric dipole moment of the PSI protein complex by a variation in the pH value of its environment.

PSI, like all proteins, is composed of amino acids, some of which are electrically charged due to the presence of side chains containing deprotonated carboxylic groups (-COO^−^) or protonated amino groups (-NH_3_^+^). The protonation state of these functional groups that are exposed on the hydrophilic regions of the PSI surface depends on the pH of the surrounding aqueous environment. At lower pH levels, an increase in protonated NH_3_^+^ groups results in a higher density of positive charges on the PSI surface. In contrast, at higher pH levels, a greater number of deprotonated COO^−^ groups contribute to an increase in negative surface charges. The overall charge distribution and net surface charge of PSI particles at a given pH are influenced by the pK values of the individual exposed COOH and NH_2_ groups which, however, are not precisely known. Thus, the theoretical estimation of charge distribution and total surface charge across the PSI surface can only be approximate for each pH condition. 

The number and type of carboxylic and amino groups exposed on its surface determine the isoelectric point, where the net surface charge of a protein is zero, i.e., when the number of positive and negative charges is equal. Under standard conditions, this characteristic is specific to each protein but can also vary depending on the composition of the solution in which the PSI particles are suspended. Previous studies have reported that the isoelectric point of plant PSI ranges from pH 4.9 to 5.0 [40], consistent with an earlier report (pH 4.5–4.8; [41]).

Importantly, the donor and acceptor sides of PSI show asymmetry in the numbers of amino and carboxylic groups on the surface, and it was calculated that the cyanobacterial PSI complex from *Thermosynechococcus elongatus* is characterized by the electric dipole moment which is almost perpendicular to the membrane plane (compare Figure 1A) and oriented from the donor to acceptor side [29] (the pH value for this calculation was not quoted). Ocakoglu et al. [36] reported net negative and positive electric potentials of the PSI–LHCI (light-harvesting complex I) complex (from the extremophilic red microalga *Cyanidioschyzon merolae*) on the donor and acceptor sides, respectively, both at pH 7 and 4.

A net positive or negative charge of PSI is a prerequisite for observing the migration of this protein in an external electric field. Such migration constitutes the basis of the method of electrodeposition of this protein on the surface of a conductive substrate and has been reported several times to be used to fabricate PSI-based biophotoelectrodes [13,29,38,39]. On the other hand, the electric dipole moment associated with the PSI particle is a prerequisite for its orientation by the electric field. In the literature, we did not find any systematic studies on the influence of different electrodeposition parameters on either (a) the efficiency of PSI migration and immobilization on the conductive substrate, (b) the orientation of PSI particles on the conductive substrate, or (c) the efficiency of photocurrent generation in photoelectrochemical cells based on PSI particles electrodeposited on a conductive substrate. Mukherjee et al. [38,39] studied the effect of PSI electrodeposition on thiol-activated gold substrates only regarding the morphology of electrodeposited PSI at different pH values of the solvent. Manocchi et al. [29] showed that electrodeposition of PSI on flattened gold electrodes was more efficient than spontaneous PSI adsorption without an electric field. However, they did not provide conclusions on the orientation of PSI particles on the substrate, and electrodeposition was only conducted at pH 7.4. Ocakoglu et al. [36] studied the effect of the pH of the sample solution (pH 4 and 7) on PSI physisorption (but not electrodeposition) on nanocrystalline n-type semiconductors, TiO_2_ and hematite. Szewczyk et al. [13] utilized PSI electrodeposition on FTO glass, again only at pH 7. 

In this paper, we characterized PSI–FTO electrodes fabricated under a range of electrodeposition conditions—including pH of the PSI solution, voltage, and duration of the electrodeposition—leading to the formation of a PSI mulitilayer composed of up to ~9 monolayers assuming homogeneous, even PSI film. Our goal was to check whether, with this method, one may control the migration direction and orientation of the PSI particles. We also aimed at testing the hypothesis that by controlling the orientation of PSI, one may control the direction of the photocurrent (cathodic vs. anodic). To realize these goals, we assumed that the total surface charge and the net electric dipole moment of PSI protein may be modulated by the pH of the PSI solution.

## 2. Results and Discussion

**Figure 3:**
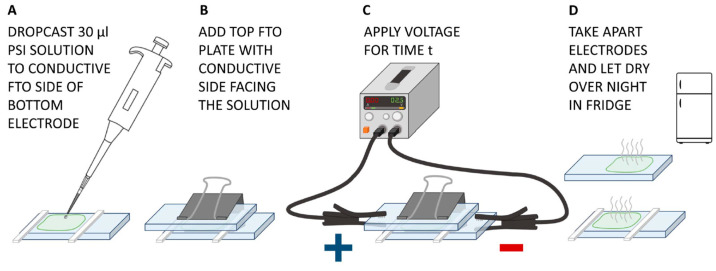
Main steps in the procedure of deposition of PSI particles on FTO conducting glass plates. (**A**) Dropcasting. (**B**) Assembling. (**C**) Electrodeposition. (**D**) Adsorption of PSI particles on the FTO plates from solution during evaporation of solvent.

The PSI–FTO working electrodes were fabricated according to the procedure summarized in Figure 3. This procedure consisted of two immobilization steps. In the first, electrodeposition step, charged PSI particles were expected to migrate towards the oppositely charged electrode—bottom or top—and to attach to it. In the second step, the PSI particles remaining in solution after electrodeposition were expected to adsorb to the FTO plates during the slow process of evaporation of the solvent.

We may speculate that during the electrodeposition step, PSI particles adhere to the FTO through the same adsorption mechanism as observed during the second step of slow solvent evaporation. The only difference is that, under an applied electric field, the migration of proteins towards the FTO surface is accelerated, occurring within a few minutes when the voltage is applied between the top and bottom electrodes.

**Figure 4:**
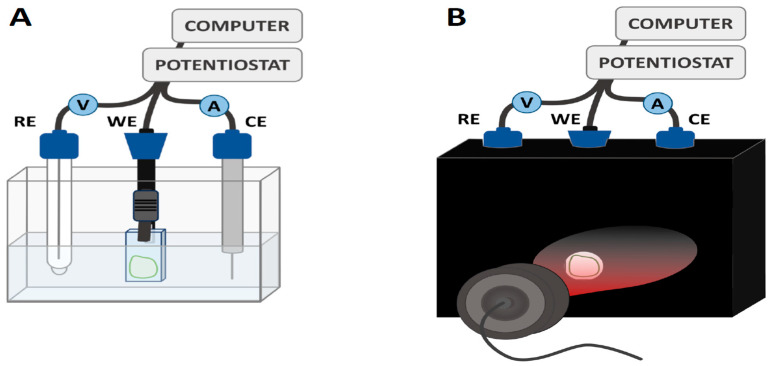
Custom-made three-electrode set-up for photochronoamperometric measurements. (**A**) PSI–FTO photoelectrode as working electrode (WE), Pt wire as counter electrode (CE), and Ag/AgCl filled with 3 M KCl (220 mV vs. SHE) as reference electrode (RE) connected to the potentiostat and measurement software. Electrodes are placed in a quartz cuvette filled with electrolyte. (**B**) Three-electrode set-up is contained in 3D-printed black housing with a hole for controlled illumination by mounted red LED at constant power.

Following the immobilization, photocurrent measurements were conducted in a three-electrode set-up shown in Figure 4 (see Section 3.4.5 for details).

### 2.1. Dependence of the Efficiency of PSI Immobilization on FTO Glass Plates on Deposition Parameters at pH 7 and 8

Generally, in electrodeposition experiments using PSI solutions at pH 7 and 8, increasing the voltage between the bottom and top FTO plates, as well as extending the deposition time, resulted in a greater amount of PSI migrating and adhering to the expected electrodes during the process.

#### 2.1.1. Absorption Spectra of PSI–FTO Plates Fabricated at pH 7

**Figure 5:**
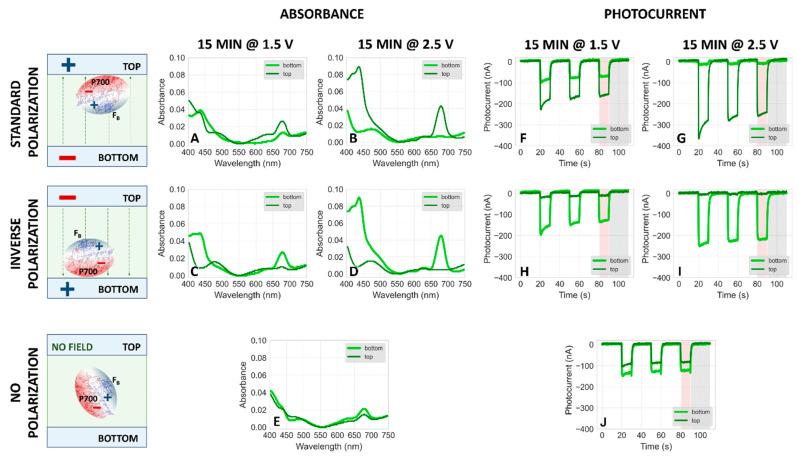
(**A**–**E**) Comparison of typical steady-state absorption spectra of PSI–FTO plates obtained by 15 min electrodeposition with standard (**A**,**B**), inverse (**C**,**D**), and no polarization (**E**) at **pH 7** followed by slow evaporation of the solvent. The light green curves represent the spectra of the bottom plates and the dark green ones the top plates. A voltage of either 1.5 V (**A**,**C**) or 2.5 V (**B**,**D**) was applied. In the case of no voltage applied, the setup—composed of two plates and a droplet of PSI suspension between them—was assembled for the same amount of time as with application of an external field. The schemes on the left side show to which FTO plates most of the PSI particles migrate and are attached, after electrodeposition and evaporation (see Figure 3), under indicated polarization conditions. (**F**–**J**) Comparison of photochronoamperometric results obtained for PSI–FTO electrodes characterized spectroscopically in panels (**A**–**E**), respectively.

Figure 5A–E demonstrates the effect of two factors on the amount of PSI particles immobilized on the top and bottom FTO plates from the solution at pH 7. One factor is the polarization of the two FTO electrodes (top electrode positive—Figure 5A,B, bottom electrode positive—Figure 5C,D, no polarization—Figure 5E; see the graphical schemes on the left side of Figure 5—in each case, the electric field is generated by the power supply providing a DC voltage) and the other one is the voltage applied between the two electrodes (1.5 V—Figure 5A,C; 2.5 V—Figure 5B,D). The efficiency of PSI deposition under these conditions may be evaluated on the basis of absorption spectra of PSI–FTO plates after completion of electrodeposition and evaporation of excess solvent. Our analysis is based only on the comparison of absorption at the Q_y_ band (~679 nm) since the blue wavelength region of the spectra is less reliable due to variable contribution from bare FTO plate background (see absorption spectrum of FTO plate in Figure S1).

For an applied voltage of 2.5 V, we observe a nearly complete migration of PSI particles towards the positively charged electrode after 15 min electrodeposition according to the data shown in Figure 5B,D. For the standard polarization, this means that almost all PSI particles are firmly attached to the top electrode (largely overcoming gravitational effects), while conversely, for the case of inverse polarization, the majority of proteins is deposited on the bottom electrode. 

The observation of PSI migration towards positive electrodes was expected since, at pH 7, negative charges prevail on this protein [40,41]. On the other hand, at lower voltage of 1.5 V (Figure 5A,C), a major part of PSI particles also migrates towards and attaches to the positively charged electrodes, but a fraction of proteins remains in solution and undergoes adsorption to the opposite FTO plate during the evaporation stage (Figure 3D), as evidenced by the small absorption peaks at 679 nm measured for the bottom plate in panel A and the top plate in panel C. 

When there is no polarization of electrodes (Figure 5E), a small fraction of PSI particles undergoes gravitational sedimentation on the bottom electrode during the 0 V “electrodeposition” stage (Figure 3C). However, the predominant fraction of PSI particles remains in the solution, clinging to the surface of each plate once they are separated when the electrodeposition setup is disassembled. During the evaporation of the solvent (Figure 3D), this fraction of PSI proteins adsorbs to their respective FTO plate, top and bottom equally. A somewhat higher efficiency of PSI attachment to the bottom than to the top electrode (as seen in a stronger absorption signal of the light green curve in Figure 5E) is explained by both the 15 min sedimentation episode as well as a somewhat larger volume of solution remaining on the bottom plate when the top gets lifted off. The gravitational sedimentation effect is also revealed by slightly higher absorbance from the negative FTO plate for the standard polarization than for inverse polarization (Figure 5A,C; see also data for pH 8 and photocurrent data discussed below). 

The data shown in Figure 5 clearly demonstrate that under the applied experimental conditions, (1) the net charge of PSI particles is negative, (2) the voltage of 2.5 V applied for 15 min is sufficient to fully control the direction of PSI migration by the electric field and attachment to either bottom or top plate, (3) the voltage of 1.5 V is insufficient for that—one fraction of PSI particles is electrodeposited on the positively charged FTO plate while another fraction is adsorbed to both plates during evaporation.

**Figure 6:**
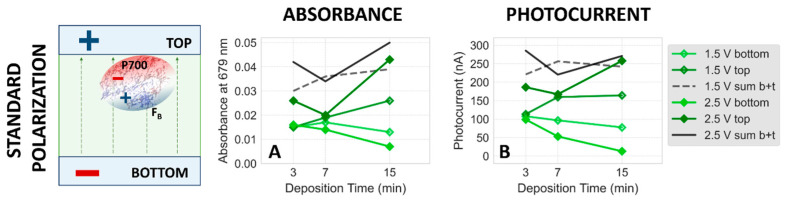
(**A**) Dependence of absorption of top, bottom, and both (sum of top and bottom) dried PSI–FTO plates on the time of electrodeposition at pH 7, for two different electrodeposition voltages (1.5 and 2.5 V), and standard polarization. (**B**) Analogous dependence of photocurrent. The photocurrent values are the average values from the shaded area of the third signal in each series (see Figure 5F–J).

Figure 6A presents the dependence of absorbance at 679 nm on electrodeposition time for standard polarization at pH 7. A general trend is observed: with increasing time of electrodeposition, the absorbance of the top plate (measured after disassembling the setup and evaporation of solvent) increases, while that of the bottom plate decreases. This trend is stronger for an applied voltage of 2.5 V and weaker for 1.5 V. This observation indicates (together with the results shown in Figure 5B) that after an electrodeposition stage of less than 15 min or performed at 1.5 V, a fraction of PSI particles remains in solution between the two FTO plates and deposits only during the evaporation stage (Figure 3D). Generally, increasing the voltage and extending the electrodeposition time result in more complete immobilization of PSI on the top electrode. Another contribution to the distribution of the PSI particles on the FTO plates is known as the “coffee ring effect”, where a fraction of PSI particles which remain in solution after the electrodeposition stage (Figure 3C) tend to “escape” from the central region of the PSI film towards the rim of the film during the evaporation stage. Such a ring is commonly observed after evaporation of the solvent and is characterized by relatively higher OD than the central part of the PSI film (Figure S2D). This might explain the observation that the total value of PSI absorbance (sum of the absorbances from the respective bottom and top plates, “b+t”) also shows a slight increase with longer deposition time. The effect is somewhat counteracted by the absorption measurement procedure where three spectra from different regions of the PSI–FTO plates are taken and the highest intensity one is typically chosen, likely including both the center and the rim parts of the deposition area.

#### 2.1.2. Absorption Spectra of PSI–FTO Plates Fabricated at pH 8

When the pH value of PSI solution is elevated from 7 to 8, a larger net negative PSI surface charge is expected and, consequently, the migration of PSI particles towards the positively charged electrode should be even more effective. The spectra collected for plates undergoing 15 min electrodeposition are very similar at the two pH values (compare Figure 5A–E and Figure S3A–E in the Supplementary Materials). However, for the data obtained for pH 8, 7 min of electrodeposition (or even 3 min at 2.5 V) were sufficient to observe maximally efficient migration of the PSI particles to the top, positively charged, while at pH 7, an increase in the electrodeposition time up to 15 min improves the efficiency of this migration (both at 1.5 and 2.5 V; compare Figure 6A and Figure S4A).

#### 2.1.3. Effect of Deionized Water

It should be emphasized that a key prerequisite for observing the migration of PSI particles toward the top electrode at pH 7 and 8 under standard polarization was the significant reduction in salt concentration of the PSI suspension used in the electrodeposition procedure. This condition was met by double dialysis of the initial stock PSI solution in deionized water with HCl-Bis-Tris buffer before electrodeposition (see Section 3). Prior experiments using distilled water in the buffer solution for dialysis, instead of deionized water, resulted in deposition of PSI mostly on the bottom electrode regardless of the polarization of electrodes. One hypothesis for this observation was a possible tendency of the proteins to aggregate in the solution with distilled water and thus their enhanced gravitational sedimentation. This was tested by several common methods of protein disaggregation prior to electrodeposition, such as ultrasound treatment by either sonication bath or rod, and rapid mixing by vortexer (see Section 3). None of these methods resulted in enhanced migration of PSI towards the top electrode. From this observation, we concluded that the ions present in distilled water efficiently screened the PSI surface charges and largely hindered protein migration in the applied electric field. 

Previous fluorescence correlation spectroscopy studies performed for freshly prepared aqueous solution of cyanobacterial monomeric PSI particles (of pH ~6.8 in the presence of 0.0006% β-DM) determined a hydrodynamic radius of 7 nm characteristic of single, non-aggregated PSI monomers [42]. This result supports our view that at pH 7 and 8 shortly after lowering the concentration of detergent, the dissolved PSI particles remain in a non-aggregated state. On the other hand, we observed gradual formation of protein sediment in the PSI solution (at low concentration of β-DM, <<0.03%) indicating that prolonged incubation of PSI particles in solution of low detergent concentration leads to their aggregation.

#### 2.1.4. Effect of Higher Voltage

We also applied a higher voltage of 3.5 V during the electrodeposition. However, such a high voltage resulted in the appearance of bubbles between the electrodes (see Figure S2B for an example), likely indicating water electrolysis and impaired PSI deposition on FTO.

At this point, one might question whether such high or even lower voltages could cause protein denaturation due to thermal effects of the applied electric field. Regarding lower voltages, this seems unlikely; previous studies demonstrated that the application of 2.5 V for 5 min during electrodeposition did not substantially affect the overall excitation dynamics in PSI–FTO plates compared to PSI in solution [42], suggesting a lack of protein degradation. Additionally, photocurrent spectra previously published for PSI electrodeposited on FTO (2.5 V for 5 min) were very similar in shape to absorption spectra of PSI in solution, further indicating the absence of protein degradation [13]. 

### 2.2. Dependence of Photocurrent Generated in the PSI–FTO Electrodes on Electrodeposition Parameters at pH 7 and 8

The correlation between the amplitude of photocurrent generated in PSI–FTO photoelectrodes and the efficiency of PSI immobilization on FTO was investigated. For this, photochronoamperometric measurements were conducted on the same PSI–FTO electrodes, which had been characterized previously by steady-state absorption (see Figure 5A–E for pH 7 and Figure S3A–E for pH 8). The corresponding photocurrents are displayed in Figure 5F–J and Figure S3F–K and generally follow the same trends as the 679 nm absorption peak signal with respect to the influence of electrodeposition voltage and polarization concerning top and bottom photoelectrodes. 

For the standard polarization conditions, the dependence of photocurrent amplitudes on electrodeposition time for both the bottom and top photoelectrodes mirrors the corresponding absorption signals at 679 nm (see Figure 6A,B, as well as Figure S4A,B). At pH 7, an increase in electrodeposition time leads to a general rise in the photocurrent generated in the top electrode (observed at both 1.5 V and 2.5 V), while the photocurrent in the bottom electrode decreases (Figure 6B), matching the trends seen in the absorption signal (Figure 6A). Similarly, at pH 8, the relationship between photocurrent and deposition time reflects the trends observed for absorbance versus deposition time (compare Figure S4A,B).

Figure 5F–J and Figure S3F–K (and others, see below) show a gradual decrease in photocurrent amplitude from the first to the third light–dark cycle. However, the amplitude stabilized in subsequent cycles. For this reason, further analysis was based on the results from the third (equilibrated) cycle. Control experiments performed with the same PSI–FTO electrodes at the beginning and end of a series of photocurrent measurements for several different electrodes in the same electrolyte yielded identical steady-state amplitudes. One factor potentially responsible for the initial change in photocurrent amplitude may be the diffusion of immobilized PSI particles from the electrode into the electrolyte solution. However, these observations indicate that, even if some PSI particles detach from the FTO plate, the detachment is not substantial. We also observed that the dried PSI–FTO electrodes retain their photocurrent generation capacity many weeks after initial use. 

#### Correlation between Absorption and Photocurrent Signals

**Figure 7:**
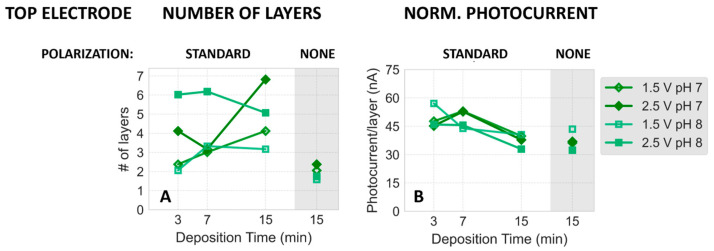
(**A**) Number of PSI monolayers on the top PSI–FTO plates computed based on absorbance at 679 nm. (**B**) Dependence of photocurrent generated in the top photoelectrodes (fabricated at pH 7 and 8 at two different electrodeposition voltages and either at standard or no polarization), normalized to the number of PSI monolayers deposited on FTO, on the time of electrodeposition. Data points in the shaded regions correspond to results obtained without applied field during deposition. Note that the two dark green and the two light green dots in the no-polarization zones should be identical (both were recorded at pH 7 and 8, respectively), so their difference represents the measurement uncertainty for these experiments.

The qualitative agreement described above between 679 nm absorbance and photocurrent signals under various electrodeposition parameters was next quantified as follows. To test whether photocurrent is proportional to the amount of PSI particles deposited on FTO, regardless of the exact pH (7 or 8) and electrodeposition time and voltage, we normalized the photocurrent (by dividing the respective photocurrent by the number of PSI monolayers deposited on FTO, which is proportional to the absorption amplitude at 679 nm—see Section 3 for calculations). Figure 7 illustrates that the normalized photocurrent remains largely unaffected by the deposition conditions, displaying only a minor decrease from approximately 50 nA per PSI monolayer at shorter electrodeposition times to around 40 nA after 15 min of electrodeposition and conditions without polarization. This observation suggests that the applied electrodeposition of PSI does not provide a significant advantage in terms of the efficiency of light-to-photocurrent conversion per PSI monolayer, compared to the spontaneous adsorption occurring during the evaporation stage. On the other hand, electrodeposition enhances PSI migration towards the expected electrodes, thus introducing strong asymmetry in PSI deposition on top and bottom electrodes, as evidenced in the data shown in Figure 5 and Figure 6 (and Figures S3 and S4). While the electric field clearly attracts the PSI particles to one of the electrodes, the lack of the effect of this field on the normalized photocurrent may be explained both by assuming (a) that it does not orient PSI particles uniformly on the FTO surface and (b) that it does.

In case (a), one may speculate that electrodeposition and spontaneous adsorption lead to equally random distributions of PSI orientations on the substrate within the multilayers. In case (b), one may assume an electric field-induced uniform orientation of the particles, which either does not increase efficiency of photocurrent generation compared to a random orientation or that spontaneous adsorption results in a similarly uniform orientation of PSI particles on the FTO surface due to some specific interactions. The former explanation (within case (b)) would be consistent with a model in which ET from the substrate to PSI occurs via the mediated mechanism (MET) rather than a direct one (DET). In this model, the direct interaction between the donor side of PSI and FTO would not play a significant role. The MET mechanism is further supported by the observation that the photocurrent signal is proportional to the number of PSI monolayers ranging from 2 to 7 (Figure 7). Thus, the monolayers farther from the FTO surface (i.e., not in direct contact) clearly contribute to the photocurrent signals, even though they are unable to support DET in any case.

It is important to note that all photocurrent signals collected for WEs fabricated from PSI solutions at pH 7 and 8 were negative, which means that electrons were transferred from FTO to PSI and subsequently via the electrolyte to CE (cathodic photocurrent). In the framework of the MET model, the specific orientation of PSI particles in the protein multilayer film does not determine the occurrence of cathodic or anodic photocurrent (compare Figure 4).

In conclusion, we are not able to determine whether PSI particles are uniformly oriented on FTO after electrodeposition at pH 7 and 8. However, our results indicate the mediated character of ET from FTO to PSI particles, and in such a case, the degree of uniformity of orientation of PSI particles is most likely not relevant. 

### 2.3. Absorption and Photocurrent Measurements Following Deposition of PSI Dissolved in Acidic Solutions (pH 4.4, 5, and 6)

#### 2.3.1. Electrodeposition at pH 6

**Figure 8:**
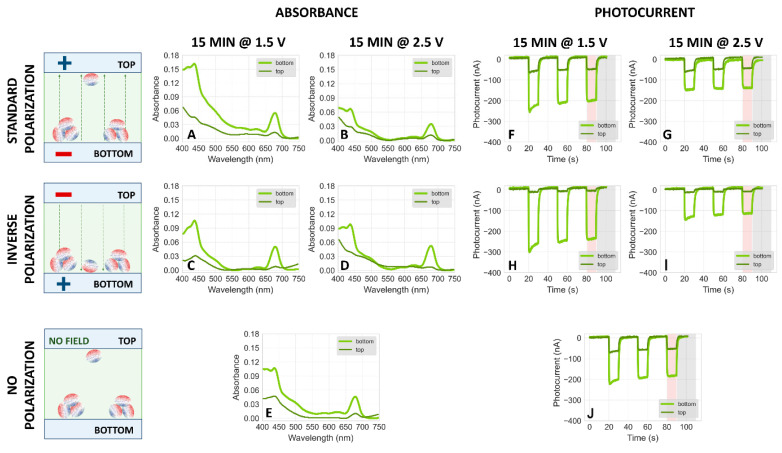
(**A**–**E**) Comparison of typical steady-state absorption spectra of PSI–FTO plates obtained by 15 min electrodeposition with standard (**A**,**B**), inverse (**C**,**D**), and no polarization (**E**) at **pH 6** followed by slow evaporation of the solvent. The light green curves represent the spectra of the bottom plates and the dark green ones represent the top plates. The voltage of either 1.5 V (**A**,**C**) or 2.5 V (**B**,**D**) was applied. In the case of no voltage applied, the setup—composed of two plates and a droplet of PSI suspension between them—was assembled for the same amount of time as those with the voltage applied. The schemes on the left-hand side show that irrespective of polarization conditions, most of the proteins stick to the bottom FTO plates after electrodeposition and evaporation (see Figure 3), but at standard polarization, some migration to the top electrode is also observed. (**F**–**J**) Comparison of photochronoamperometric results obtained for PSI–FTO electrodes characterized spectroscopically in panels (**A**–**E**), respectively.

Figure 8 compares the absorption spectra and photocurrents of PSI particles electrodeposited at pH 6, under the same conditions as data collected at pH 7 and 8 (see Figure 5 and Figure S3). The results indicate that the majority of proteins migrate to the bottom FTO plate, regardless of polarization during electrodeposition. This suggests either significant protein aggregation leading to gravitational sedimentation or ineffective electrophoretic migration due to the too small net charge of the protein complexes at pH 6. The former is more plausible, given that both absorption and photocurrent signals are markedly higher on the bottom plate under no polarization (Figure 8E,J), compared to only minor differences observed in samples at pH 7 and 8 (Figure 5E,J and Figure S3E,K). This difference demonstrates that shortly after assembling the bottom and top FTO plates into a sandwich system (see Figure 3B,C), the majority of PSI particles at pH 6 underwent gravitational sedimentation onto the bottom plate. This behavior contrasts with that observed at pH 7 and 8, where the distribution of particles was more uniform. Furthermore, under standard polarization conditions (1.5 V and 2.5 V, Figure 8A,B,F,G), signals from the top plates are relatively stronger than under inverse polarization (Figure 8C,D,H,I), indicating some non-aggregated proteins migrating towards the positively polarized top electrode, consistent with a net negative charge (as seen for pH 7 and pH 8). This observation was corroborated by an independent experiment where, prior to the fabrication of the PSI–FTO electrode, the PSI solution underwent a 2 min disaggregation in a sonication bath. The results, shown in Figure S5, demonstrate that at pH 6, the disaggregated proteins migrated towards the positive electrode. These findings indicate that the isoelectric point of the investigated PSI particles is below 6.

**Figure 9:**
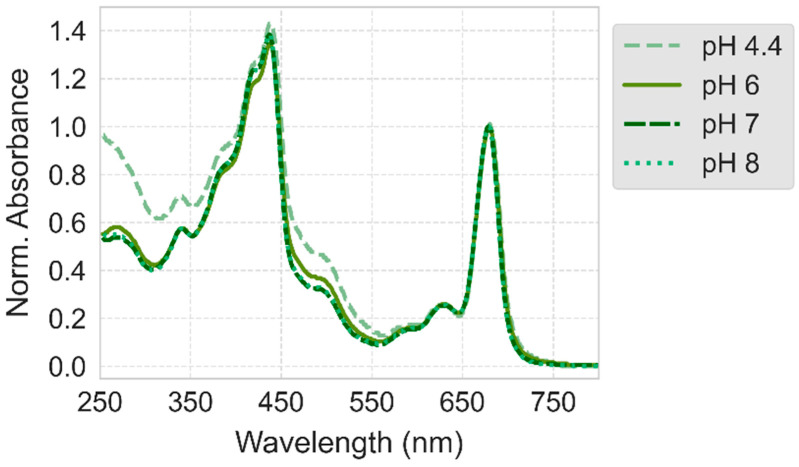
Steady-state absorption spectra of PSI solutions at four different pH values. Note that the PSI proteins investigated at pH 4.4 were stored frozen with glycerol. The spectra of the remaining PSI solutions were recorded for PSI proteins stored frozen without glycerol. PSI solutions at pH 7 and 8 contained Bis-Tris buffer while those at pH 4.4 and 6 contained MES buffer (see Section 3).

PSI aggregation at pH 6 was reported before [40]. It seems unlikely that the reason for this aggregation is denaturation of PSI leading to exposition of hydrophobic parts of the protein complex—many earlier studies reported a high degree of stability of PSI at pH 6 (see for example [43]). On the other hand, the steady-state absorption spectrum of PSI solution recorded at pH 6 was slightly different from those at pH 7 and 8 (Figure 9), but the reason for that may be the different composition of the buffer solution (see Section 3). A more plausible explanation for aggregation is that pH 6 is close to the isoelectric point of PSI. At the isoelectric point, the overall charge is zero and hence repulsive interactions between charged proteins are expected to be weak, whereas dipole–dipole attractive interactions prevail due to the substantial electric dipole moment between the positively and negatively charged sides of the complex. In order to further investigate the problem of aggregation and the isoelectric point of PSI complexes and, more importantly, to check the possibility of depositing proteins on FTO with inverse orientation (“F_B_”-side facing the FTO surface) and generating anodic photocurrent, we performed a series of experiments at pH 4.4 and 5. 

#### 2.3.2. Electrodeposition at pH 4.4 and 5

**Figure 10:**
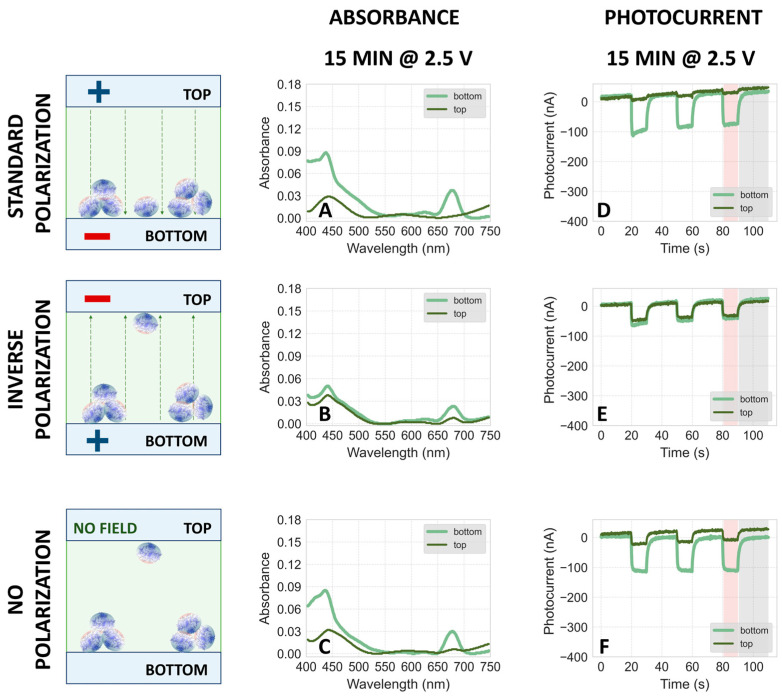
(**A**–**E**) Comparison of typical steady-state absorption spectra of PSI–FTO plates obtained by 15 min deposition at 2.5 V with standard (**A**), inverse (**B**), and no polarization (**C**) at **pH 4.4**. The light green curves represent the spectra of the bottom plates and the dark green ones represent the top plates. A voltage of 2.5 V was applied. In the case of no polarization, the setup—composed of two plates and a droplet of PSI suspension between them—was assembled for the same amount of time as those with the voltage applied. The schemes on the left-hand side show to which FTO plates most of the PSI particles migrate and are attached, after electrodeposition and evaporation (see Figure 3), under the indicated polarization conditions. (**D**–**F**) Comparison of photochronoamperometric results obtained for PSI–FTO electrodes characterized spectroscopically in panels (**A**–**C**), respectively.

Figure 10 presents the absorption spectra and photocurrents for PSI particles electrodeposited at pH 4.4. Similar to the results at pH 6, the signals from the bottom electrodes are consistently stronger than those from the top—regardless of polarization during electrodeposition—suggesting significant aggregation. This aggregation is attributed to the denaturing effect of the low pH, as supported by the distinct absorption spectrum at this pH compared to others (Figure 9). Data from inverse polarization (Figure 10B,E) reveals a small fraction of (likely non-aggregated) proteins migrating electrophoretically to the negatively charged top electrode, suggesting that at pH 4.4, PSI particles possess a net positive charge, indicating that the isoelectric point is above this pH. Notably, despite the likely inverse orientation of PSI complexes on the top FTO plate (with the acceptor side facing the substrate, compare Figure 2), there is no resultant anodic photocurrent, reinforcing the notion that the direct interaction between PSI and FTO is not the primary determinant of photocurrent direction in these systems.

The electrophoretic migration results indicate that the isoelectric point of the PSI particles is between pH 4.4 and 6. To refine this range, further studies were conducted at pH 5, as shown in Figure S6. These results closely resemble those at pH 4.4, suggesting that the isoelectric point of the PSI proteins lies between pH 5 and 6. This finding helps narrow down the precise pH range where the net charge of the PSI particles is zero.

### 2.4. Theoretical Predictions of PSI Surface Charges

**Figure 11:**
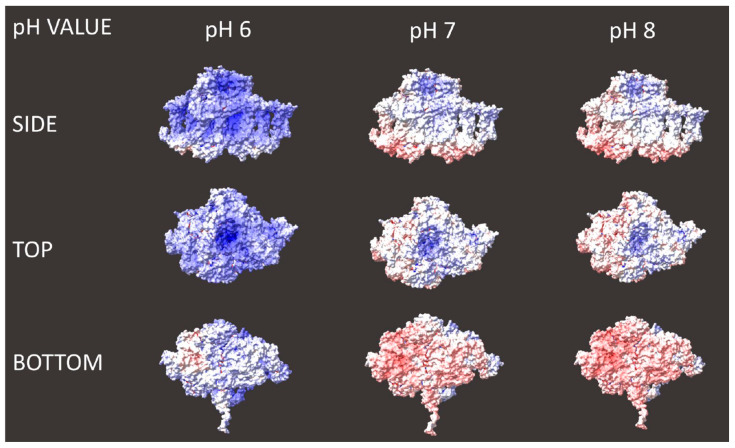
Dependence of PSI surface charge distribution on pH of PSI solution. Red (blue) color represents negative (positive) charges.

In order to predict the theoretical value of the isoelectric point and compare it to the experimental results, we performed computational simulations of the distribution of surface charges of this protein complex (based on the known molecular structure of PSI [44]) in solutions at different pH (see Section 3 for details). Figure 11 shows the results of these computations—three projections of PSI (side, top or stromal, and bottom or luminal) at three pH values relevant to our experimental studies. According to these simulations, the amount of negative and positive charges is best balanced at pH 7, while at pH 6 and 8 positive and negative charges predominate, respectively. These results indicate that the expected value of the isoelectric point is about pH 7. Indeed, a computation based on the solved monomeric PSI structure (PDB ID 6HQB) by the Prot Pi Protein Tool yields values for the isoelectric point ranging from 6.6 to 6.9 depending on the chosen data source for pK values, while a similar result of about 6.7 is obtained using the Expasy tool by the Swiss Institute of Biotechnology. However, these values are significantly higher than our experimental data suggest (between pH 5 and 6). One reason for this discrepancy may be that the simulations cannot fully represent the effect of the presence of counterions which may screen the surface PSI charges to some extent. Although we used deionized water in the experiments, the pH of the applied buffers was adjusted by using either HCl (to reach pH 7 and 8) or NaOH (to reach pH 6). Another reason may be the belt of detergent which may hinder the flow of protons between the solution and hydrophobic parts of the protein’s surface.

On the other hand, our simulations show that the negative charges are mostly associated with the donor side of PSI (“bottom”), while positive charges are located on the acceptor side (“top”), in accordance with earlier reports [29,36]. These results would indicate the presence of an electric dipole moment associated with the PSI particle, directed from the donor to the acceptor side of this complex. Thus, our computational simulations indicate a possibility of orienting PSI complexes by an external electric field during the electrodeposition. 

### 2.5. Characterization of the PSI–FTO Surface by Optical Microscopy

**Figure 12:**
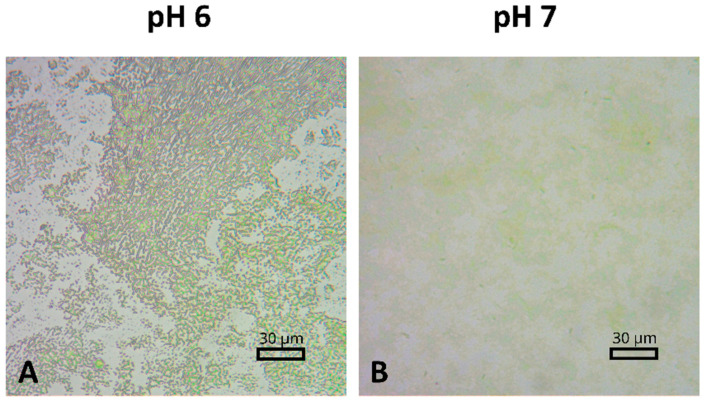
Comparison of optical microscope images of PSI–FTO plates fabricated at pH 6 (**A**) and pH 7 (**B**).

Figure 12 presents optical microscopy images of PSI–FTO plates fabricated at pH 6 and pH 7. The images demonstrate that the PSI film deposition on the FTO layer is heterogeneous under both conditions. At pH 7, the PSI proteins aggregate into patches with varying optical densities. This variability suggests that the average thickness and absorbance of the PSI films, as determined in the preceding experiments, are influenced by regions of differing thickness and absorbance. At pH 6, where PSI proteins tend to aggregate more strongly, the microscopic image reveals even greater complexity with diverse microstructural features (Figure 12A). The variety of microstructures observed at pH 4.4 and 5 is even richer. The origins of these phenomena are obscure and worthy of further investigation.

One of the arguments supporting the mediated character of ET from FTO to PSI in our considerations above is based on the observation that most of the photocurrent is generated in PSI proteins that do not directly interact with FTO. However, the presence of microscopic patches/structures with varying thickness in the PSI film raises questions regarding the specific regions where photocurrent generation occurs. Based on the approach described in Section 3, the internal quantum efficiency (IQE) for the highest photocurrents reported in this paper (~250 nA or ~400 nA/cm^2^) was estimated at ~0.3%. It could be argued that with such a low IQE, photocurrent generation may be restricted to the regions of the PSI film that are devoid of the green coloration, as depicted in Figure 12. These patches are likely to contain only a single monolayer of PSI proteins directly interacting with the FTO surface. The observed proportional dependence of photocurrent on the average number of monolayers does not necessarily invalidate this hypothesis. Instead, it is plausible that, as the total amount of PSI on the FTO increases, the colorless patches initially sparsely populated with the proteins become progressively more populated with additional PSI proteins, eventually forming more complete but still colorless monolayers. Thus, the photocurrent generation could be confined to these increasingly populated monolayer regions.

However, this argument may be weakened by prior observations: (a) only 4% of monomeric PSI in solution, when utilizing the same mediators (ascorbate and DCPIP), contributes to photocurrent generation, while 96% of PSI undergoes charge recombination [45], and (b) following deposition of PSI particles on FTO, approximately 20% of the 4% of PSI that can contribute to the photocurrent is permanently oxidized (P700^+^), rendering the particles unable to generate photocurrent [46]. Consequently, it may be estimated that only about 0.8% of PSI particles is available to contribute to the photocurrent in the PSI–FTO electrode, a value not very different from the observed IQE of ~0.3%. It is reasonable to assume that this 0.8% of functional PSI particles is distributed randomly across both “green” and “colorless” patches rather than being confined almost exclusively to the latter. Therefore, we propose that the photocurrent observed under the various conditions reported in this study is predominantly generated in the multilayered, green patches of PSI proteins that do not directly interact with the FTO surface. This conclusion is supported by independent modeling of experimental results as reported in [46]. Hence, mediated electron transfer (MET) appears to be a more plausible mechanism than direct electron transfer (DET) in our systems.

## 3. Materials and Methods

### 3.1. Preparation of PSI Particles

The preparation of PSI solutions followed the procedure outlined in [42]. Briefly, *Synechocystis* sp. PCC 6803 cell cultures were grown in BG-11 medium under continuous white light and stirring. Cells were harvested by centrifugation in the log phase of growth. Next, rod sonication was performed in the presence of lysozyme and protease inhibitors to break cell walls and protect protein complexes, respectively. The PSI complexes were extracted from the obtained thylakoids using *n*-dodecyl-*β*-D-maltoside (*β*-DM) detergent and purified by ion-exchange chromatography, with a weak anion exchanger resin and MgSO_4_ gradient elution. Fractions with specific absorption characteristics (absorption maximum ~679 nm) were pooled and concentrated, and samples were resuspended in a fresh buffer containing 20 mM Bis–Tris (pH 7.0), 5 mM MgCl_2_, 5 mM CaCl_2_, 10 mM NaCl, and 0.03% β-DM (*v*/*v*) before storage at −20 °C until further use. The PSI solutions were stored at −20 °C either in the presence of 20% glycerol (proteins used for electrodeposition at pH 4.4, 5, 6, 7, and 8) or without glycerol (proteins used for electrodeposition at pH 6, 7, and 8). Respective results obtained for the proteins stored under both conditions did not show significant differences. Results presented in this paper were obtained from the proteins stored without glycerol (all data at pH 7 and 8 and some data at pH 6) and with glycerol (all data at pH 4.4 and 5 and some data at pH 6). 

To reduce detergent and salt concentrations, which is necessary for electrodeposition, and to achieve PSI solutions with varying pH values, frozen PSI samples were diluted to OD_1cm,679nm_ = 3–5 in 1 mL aliquots and dialyzed using Spectra-Por Float-A-Lyzer G2 filters. Dialysis filters were washed with a 10% ethanol solution for 20 min, followed by water (distilled or deionized) before loading with the PSI solution. Buffer exchange occurred in 500 mL of buffer solution (prepared usually using deionized water, except for some experiments described in Section 2.1.3 “Effect of deionized water”, for which distilled water was used), under constant slow stirring (150 rpm) at 6 °C for 2 days. Subsequently, the buffer was exchanged for fresh 500 mL for another 2 days of dialysis. For pH values of 7 and 8, we used 7.5 mM Bis–Tris with pH adjusted using 5 M HCl, while 5 mM MES buffer titrated with 5 M NaOH was used for samples at pH 6 and lower. The resulting PSI solution was diluted with distilled or deionized water, to reach an OD of 1 at the 679 nm absorption peak (in a 1 cm thick cuvette), and used in electrodeposition experiments.

### 3.2. Disaggregation Methods of PSI Aggregates

In the standard procedure, the dialyzed and diluted PSI solution was used with no other disaggregation steps apart from gentle shaking of the Eppendorf tube containing the sample. Since in some experiments, electrophoretic migration of PSI particles was hardly observed, we tried to induce it by disaggregation of possible protein aggregates likely responsible for this observation. Different disaggregation methods were tested. For this, 70 µL aliquots of the PSI solution were separated for each cycle and either vortexed for 2 min with short breaks or sonicated for 2 min on ice in a sonication bath or homogenized by rod sonication on ice for 3 s total in 1 s intervals with 10 s breaks at 20% power setting (Sonics VibraCell VCX130 from Sonics & Materials, Inc., Newtown, CT, USA). Disaggregation was carried out immediately before deposition to limit any reaggregation to a minimum.

### 3.3. Preparation of the PSI–FTO Electrodes

Fluorine-doped tin oxide coated glass (FTO conducting glass, FTO plates), purchased from Sigma Aldrich, featuring a surface resistivity of 13 Ω/sq and a thickness of 2.2 mm, served as the substrate for the photoelectrodes. Larger slides were cut into pieces measuring 1 cm in width and 2.5 cm in length. The substrate was thoroughly washed using a sonication bath, initially with soap and then twice with ethanol. The conductive side was identified, electrodes were labeled, and designated bottom plates were equipped with two narrow spacers approximately 1 mm thick, made from 10 layers of tape.

The deposition steps were performed at room temperature (except for the last evaporation step) and are illustrated in Figure 3. (A) A drop of 30 µL of PSI solution was deposited onto the conductive surface of the bottom electrode and spread close to the edges and spacers while avoiding actual contact. (B) The top FTO electrode was then placed on top (with the conductive side facing the solution). (C) The bottom electrode was connected to the negative contact of the power supply (NDN, model DF173005C), while the top electrode was connected to the positive contact—this is called standard polarization. The opposite polarization with the positive electrode as bottom plate is denoted as inverse. Reference samples without applied potential were also fabricated. The selected voltage setting of the applied potential was 1.5 V or 2.5 V for varying time periods between 3 min and 15 min. Two depositions with identical DC power supplies were conducted in parallel. (D) After the electrodeposition time, the two glass plates were separated and excess solution was left on the surfaces to dry. For that, the FTO plates were placed in the fridge at ≈4 °C overnight for the solvent to evaporate. The surface area of the PSI film, which was derived from photographs of the dried photoelectrodes, was found to be slightly smaller on average for the analyzed top electrodes compared to the bottom ones yielding values of 0.6 ± 0.03 cm^2^ and 0.65 ± 0.03 cm^2^, respectively.

### 3.4. Characterization of the Photoelectrodes

Dried photoelectrodes were characterized in terms of the distribution of the amount of deposited PSI with respect to top and bottom electrodes (using steady-state absorption spectroscopy), the uniformity of each film (observed by optical microscopy), as well as their performance at generating photocurrent. Both absorption and photocurrent experiments were conducted at room temperature. 

#### 3.4.1. Steady-State Absorption Spectroscopy

Steady-state absorption UV–visible spectra were measured using a Hitachi U-2800A spectrophotometer (Hitachi, Ltd., Tokyo, Japan). A minimum of three spectra was acquired for each photoelectrode at different positions to accommodate possibly uneven deposition. Spectra were baseline-subtracted by a measurement in air and offset-corrected (by subtraction of the minimum value over the entire wavelength region). The best spectrum was selected for each sample, representing a balance between data quality and highest absorbance at the Q_y_ band maximum.

#### 3.4.2. Estimation of the Average Number of PSI Monolayers Deposited on FTO

For further analysis, the absorbance at 679 nm, *A*_679nm_, was extracted and following [13], the approximate average number of densely packed PSI monolayers *n_PSI_* forming the obtained multilayer was computed according to Equation (1):

(1) $$n_{PSI}=\frac{A_{679nm\;\cdot N_{A}\cdot d^{2}}}{\varepsilon_{679nm}\cdot m},$$

where *N_A_* is Avogadro’s constant, *d* is the height of the PSI complex (which is assumed to be spherical) and is assumed to be 12 nm based on [47,48], *ε*_679nm_ is the molar extinction coefficient of chlorophyll *a* in PSI (57,000 (Mcm)^−1^ according to [49]) and *m* is the number of chlorophylls in a single monomeric PSI (which is 96 according to [50]). According to this formula, absorbance *A*_679nm_ = 0.0063 corresponds to a single monolayer of densely packed PSI particles. Consequently, the PSI films on the photoelectrodes fabricated for the presented experiments are composed of up to 9 monolayers (corresponding to *A*_679nm_ = 0.057). 

#### 3.4.3. Estimation of Internal Quantum Efficiency

Internal quantum efficiency (IQE) is defined as the ratio of the number of photoelectrons contributing to the photocurrent and the number of photons absorbed by PSI particles. As shown in detail in [13], IQE may be estimated on the basis of the LED emission spectrum, the absorption spectrum of PSI deposited on FTO, and the amplitude of photocurrent density. 

#### 3.4.4. Optical Microscopy

The microscopic structure of the dried PSI films was investigated using an optical microscope (Evolution 100 from Delta Optical, Nowe Osiny, Poland) at 10× and 40× magnification. 

#### 3.4.5. Photochronoamperometric Measurements

For photocurrent measurements, a custom-made three-electrode photo-electrochemical cell (PEC) was used as depicted in Figure 4 (compare [13]) and is described as follows.

In Figure 4A, the photoelectrode—PSI immobilized on an FTO plate—is connected as the working electrode (WE) in the center of the set-up. A platinum wire serves as the counter electrode (CE) to measure both the dark currents and photocurrents generated in WE. The electric potential applied to WE is controlled against a reference electrode (RE) consisting of Ag/AgCl in 3 M KCl (220 mV vs. SHE). Data acquisition is managed by the Autolab PGSTAT204 potentiostat (from Metrohm Autolab BV, Utrecht, Holandia) and Nova Metrohm software (version No. 1.11). Before photocurrent generation, the open circuit potential (OCP) was determined in the dark (after a few minutes of equilibration) and then applied to each working electrode throughout the measurement protocol in order to maintain a zero value for the dark current between CE and WE. The electrolyte was freshly mixed for each sample series (up to 45 individual photoelectrodes in a row) to achieve final concentrations of 30 mM Bis–Tris buffer (pH 7), 10 mM sodium ascorbate, and 200 µM dichlorophenolindophenol (DCPIP). This set of ET mediators was successfully used with PSI particles to generate photocurrent in several biohybrid solar cell systems [13,27,51].

Figure 4B shows that the spectroelectrochemical cell is fitted with a lid and completely enclosed by a black box to block out ambient light, ensuring controlled dark and illuminated conditions. A 685 nm red light-emitting diode (LED) is mounted in front of a hole in the box at a constant distance as the illumination source, which was previously characterized with a light power density of 5.8 mW/cm^2^ and approximately 24 nm wide spectral band (FWHM) [13,45]. The LED is operated through an automated program consisting of three cycles. Each cycle comprises a 20 s period of darkness followed by 10 s of illumination. The effective photocurrent is derived from the difference between photocurrent measured in dark (current close to zero due to application of OCP to WE) and illuminated periods. The data are cleaned from outliers using the 2 percentile approach before averaging the respective plateaus and subtracting them (8 s contributing to dark values and 6 s to illuminated values; indicated by shaded areas in the photocurrent plots—see below). The shown data correspond to the final cycle, approaching a steady state. 

### 3.5. Computation of Surface Charges

In order to predict the behavior of PSI particles (migration and orientation) in solutions of different pH values in the presence of an electric field generated during the electrodeposition, the electrostatic surface potential of PSI was computationally modeled. As a reference value, the isoelectric point was computed based on the amino acid sequence of cyanobacterial PSI from monomeric *Synechocystis* sp. PC 6803 from the solved crystal structure that can be found in the RCSB Protein Data Bank under the PDB ID 6HQB [43]. Results found by the Prot Pi Protein Tool using the original FASTA sequences of the 11 subunits yielded values for the isoelectric point ranging from 6.6 to 6.9 depending on the chosen data source for pK values (Expasy or Native, respectively). Using the entire sequence without separate subunits with Expasy’s own computation tool by the Swiss Institute of Biotechnology returns a similar value of 6.67.

The isoelectric point provides a first estimate of the net charge of PSI at a given pH. For further insights on how this charge is distributed across the surface of the pigment-protein complex, the electrostatic potential was computed using the Adaptive Poisson-Boltzmann Solver (APBS) algorithm. The PDB file was prepared by fixing all missing heavy atoms and incomplete residues from the structure. With the help of the Protein Science server, this was done in two separate parts that were ultimately joined back together—this proved necessary due to the large size of the structure. Next, the PDB file was converted into a PQR file using the APBS server’s PDB2QR tool, where charges and radii are assigned based on force field parameters according to the chosen pH values (6, 7, and 8). The obtained PQR file acts as input for the APBS calculation using default parameters. APBS computes the electrostatic properties of each protein subunit according to the Poisson–Boltzmann equation yielding a .dx output file. Using UCSF ChimeraX (version 1.6.1) visualization software, these could be mapped to the surface of the original structure 6HQB, rendering positive charges in blue and negative charges in red ranging from +25 to −25 kT.

Computational tools used in the course of this work: 

RCSB Protein Data Bank https://www.rcsb.org/ (accessed on 30 July 2024) [52].

Expasy https://web.expasy.org/compute_pi/ (accessed on 30 July 2024) [53,54].

Prot Pi Protein Tool https://www.protpi.ch/ Release: 2.2.29.152 (accessed on 30 July 2024).

APBS https://server.poissonboltzmann.org/ (accessed on 30 July 2024) [55].

Protein Science https://www.protein-science.com/ (accessed on 30 July 2024) [56].

UCSF ChimeraX https://www.cgl.ucsf.edu/chimerax/ (accessed on 30 July 2024) [57].

Matplotlib https://matplotlib.org/ (accessed on 30 July 2024) [58].

Pandas https://pandas.pydata.org/ (accessed on 30 July 2024) [59].

## 4. Conclusions

Different experimental conditions were applied to immobilize PSI particles on the FTO glass to fabricate PSI–FTO photoelectrodes, using the method of electrodeposition. These experiments provide deeper insight into the processes involved in the fabrication and generation of photocurrent in the PSI–FTO-based photoelectrochemical cell. The effect of immobilization was tested by steady state absorption and photocurrent measurements, and complemented by optical microscopy. We have shown that the total PSI surface charge may be changed from negative to positive by adjusting the pH of the solution (ranging from 8 to 4.4, respectively). Thus, at a given pH, the direction of protein migration during electrodeposition may be controlled by the direction of the electric field. Unfortunately, at acidic pH, between 4.4 and 6, PSI particles largely aggregate, impairing the efficiency of electrophoretic migration of the proteins. It remained unclear whether the electric field may also control the orientation of PSI on the FTO substrate, since regardless of the pH of the PSI solution (and intended PSI orientation on FTO), the electron transfer was always cathodic—from FTO to PSI. Moreover, the electrodeposition did not substantially increase the efficiency of photocurrent generation compared to the control PSI–FTO electrodes fabricated by the simple drop-casting method followed by slow evaporation of the solvent. The only parameter that clearly affected the net photocurrent was the optical density of PSI film on FTO. This conclusion, together with the observation of only cathodic photocurrent, points to the mediated character of electron transfer from FTO to PSI particles, forming, on average, a multilayer composed of up to 9 monolayers of densely packed proteins. Optical images of the PSI–FTO surface show that the structure of PSI film is not homogeneous; rather, flat patches of clustered proteins are observed, and this effect is more evident for electrodes fabricated at lower pH values. The IQE of fabricated PSI–FTO electrodes does not exceed 0.3%. We conclude that this value is related to the specific composition of electrolyte (10 mM sodium ascorbate and 200 μM DCPIP) and the design of our PSI–FTO-based photoelectrochemical cell, which together determine the coupling of the donor and acceptor sides of PSI with ET mediators.

## Data Availability

Data is contained within the article and Supplementary Materials.

## Supplementary Materials

The following supporting information can be downloaded at: https://www.mdpi.com/article/10.3390/ijms25189772/s1.
