# Supplementary material for: Dependence of Protein Immobilization and Photocurrent Generation in PSI–FTO Electrodes on the Electrodeposition Parameters
Source: Int J Mol Sci. 2024 Sep 10;25(18):9772. doi: 10.3390/ijms25189772 (PMC11431872; doi:10.3390/ijms25189772)
Supplement: Supplementary file 1 [file ijms-25-09772-s001.zip › ijms-3161169-supplementary.pdf]

Supplementary Materials

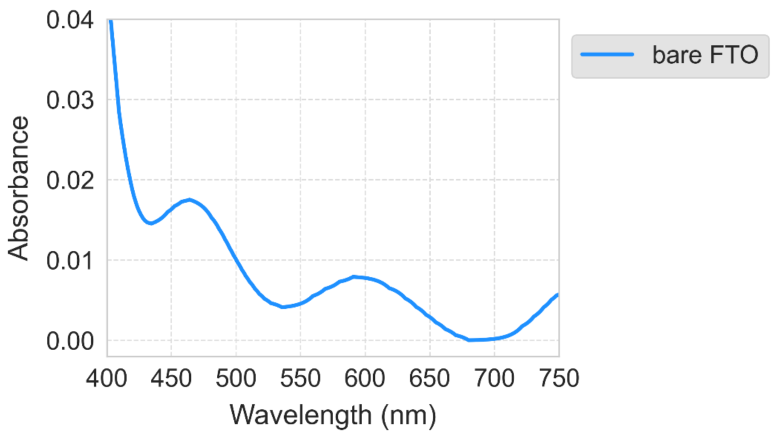

Figure S1. Steady-state absorption spectra of bare FTO plate

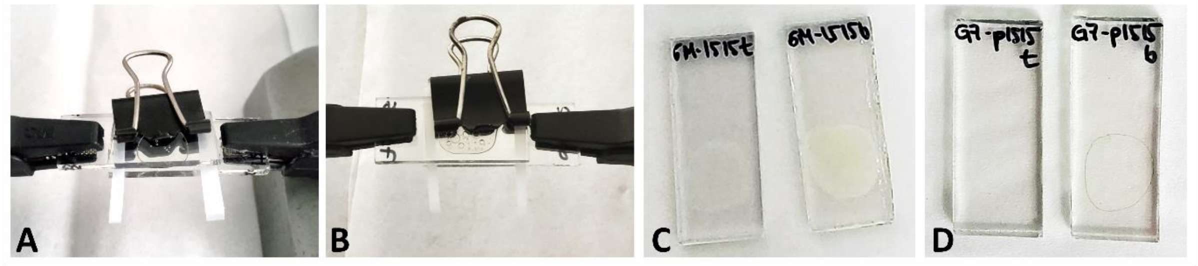

Figure S2. (A) Photoelectrodes during electrodeposition with PSI solution sandwiched between top and bottom FTO plates. (B) Photoelectrodes during electrodeposition at higher voltage of 3.5 V with PSI solution forming bubbles. (C) PSI-FTO electrodes after drying with a uniform PSI film on both top and bottom plates. (D) PSI-FTO electrodes after drying with an uneven distribution of PSI complexes between top and bottom plates and formation of a darker rim.

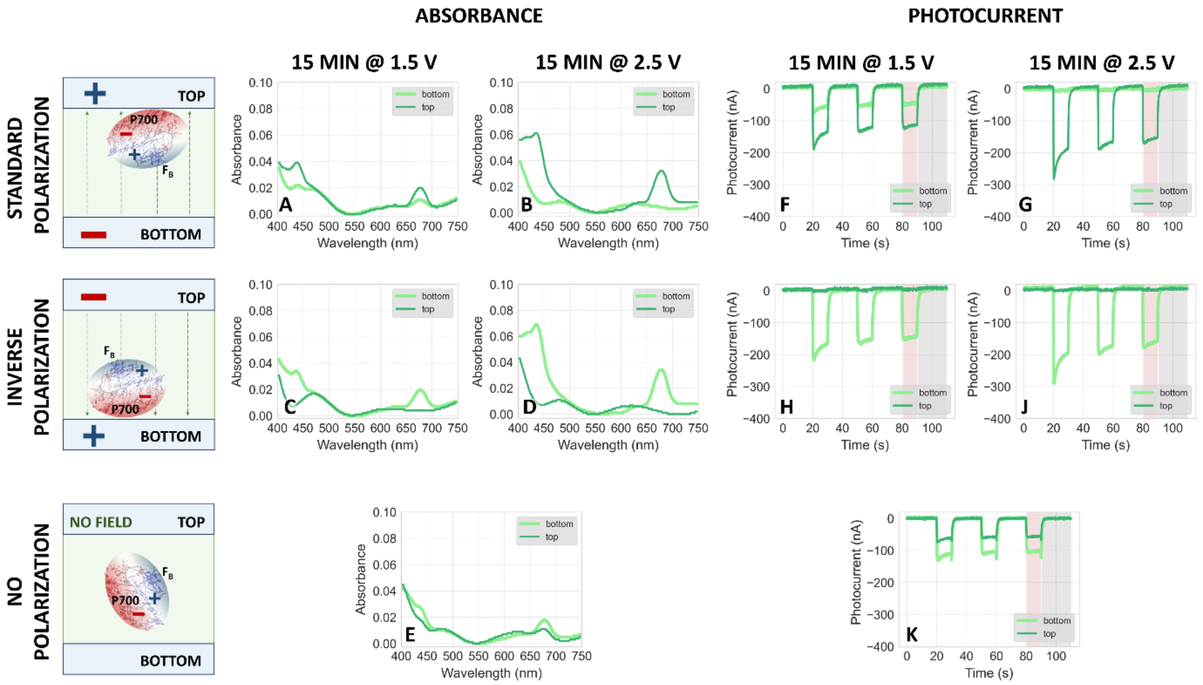

Figure S3. (A-E) Comparison of typical steady-state absorption spectra of PSI-FTO plates obtained by 15 minutes electrodeposition with standard (A-B), inverse (C-D) and no polarization (E) at **pH 8** followed by slow evaporation of the solvent. The light green curves represent the spectra of the bottom plates and the dark green ones represent the top plates. The voltage of either 1.5 V (A, C) or 2.5 V (B, D) was applied. In the case of no voltage applied, the setup – composed of two plates and a droplet of PSI suspension between them – was assembled for the same amount of time as those with the voltage applied. The schemes on the left-hand side show to which FTO plates most of the PSI particles migrate and are attached, after electrodposition and evaporation (see Fig. 2), under the indicated polarization conditions. (F-K) Comparison of photochronoamperometric results obtained for PSI-FTO electrodes characterized spectroscopically in panels A-E, respectively.

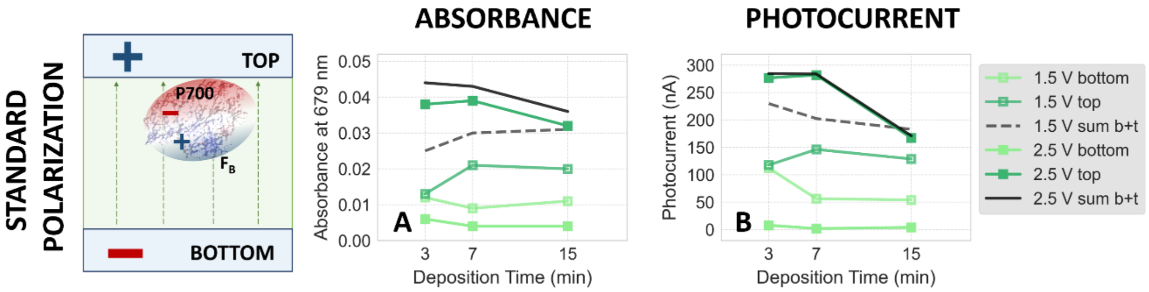

Figure S4. (A) Dependence of absorption of top, bottom, and both (sum of top and bottom) dried PSI-FTO plates on the time of electrodeposition at **pH 8**, for two different electrodeposition voltages (1.5 and 2.5 V), and standard polarization. (B) Analogous

dependence of photocurrent. The photocurrent values are the average values from the shaded area of the third signal in each series (see Fig. S1F-K).

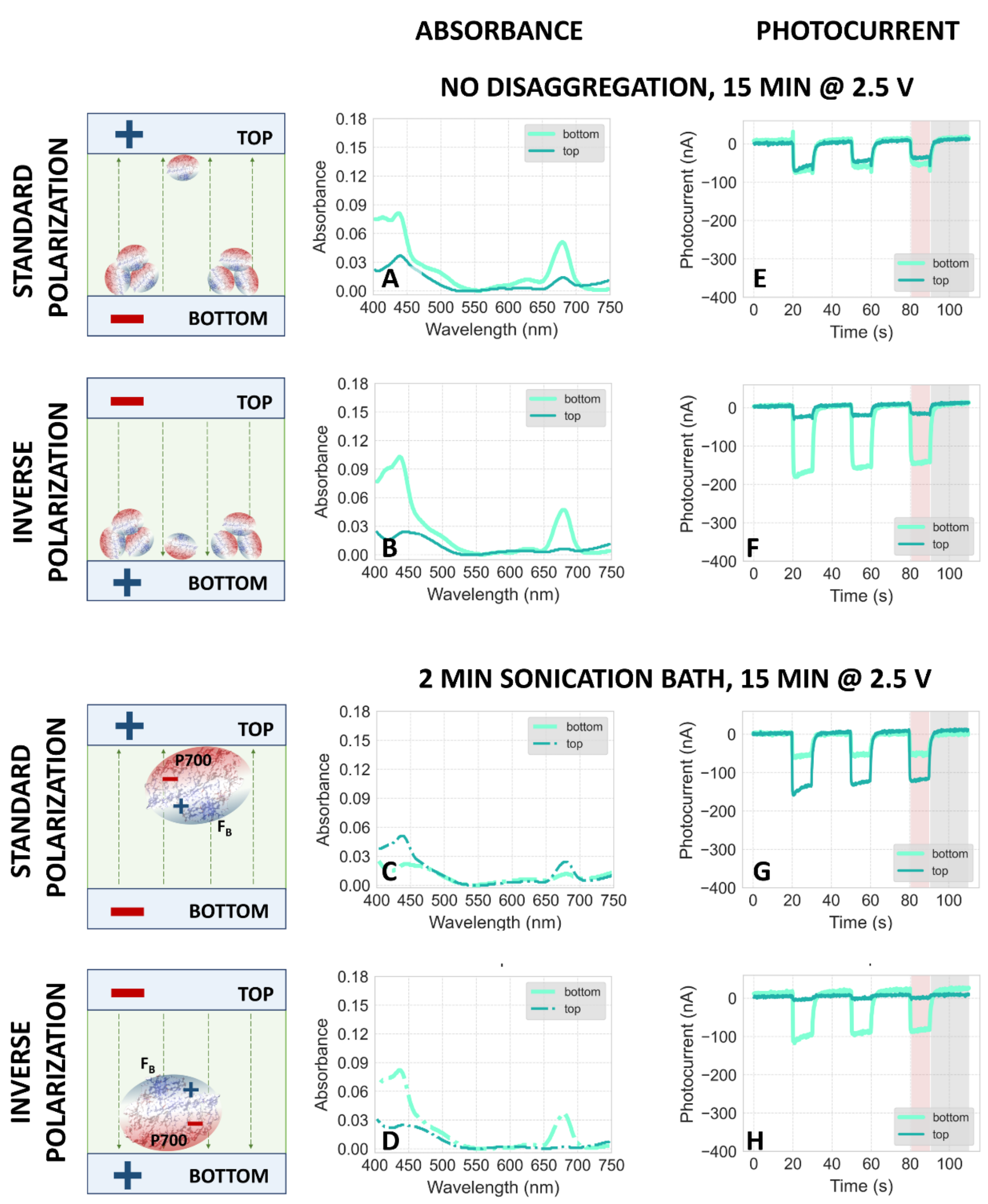

Figure S5. Effect of disaggregation of PSI aggregates formed at **pH 6**. (A,B) Absorption spectra from the bottom and top PSI-FTO plates fabricated by 15 minutes electrodeposition at 2.5 V without any PSI disaggregation procedure, at standard and inverse polarization, respectively. (C,D) Absorption spectra from the bottom and top PSI-FTO plates fabricated as in panels A and B, respectively, but with PSI solution that was subjected to a procedure of 2 minutes disaggregation in a sonication bath. (E-H) Photocurrents recorded for the PSI-FTO electrodes whose absorption spectra are shown in the respective panels A-D. (Note that the PSI

proteins used in this experiment were stored with glycerol, unlike those used in the experiment shown in Fig. 8).

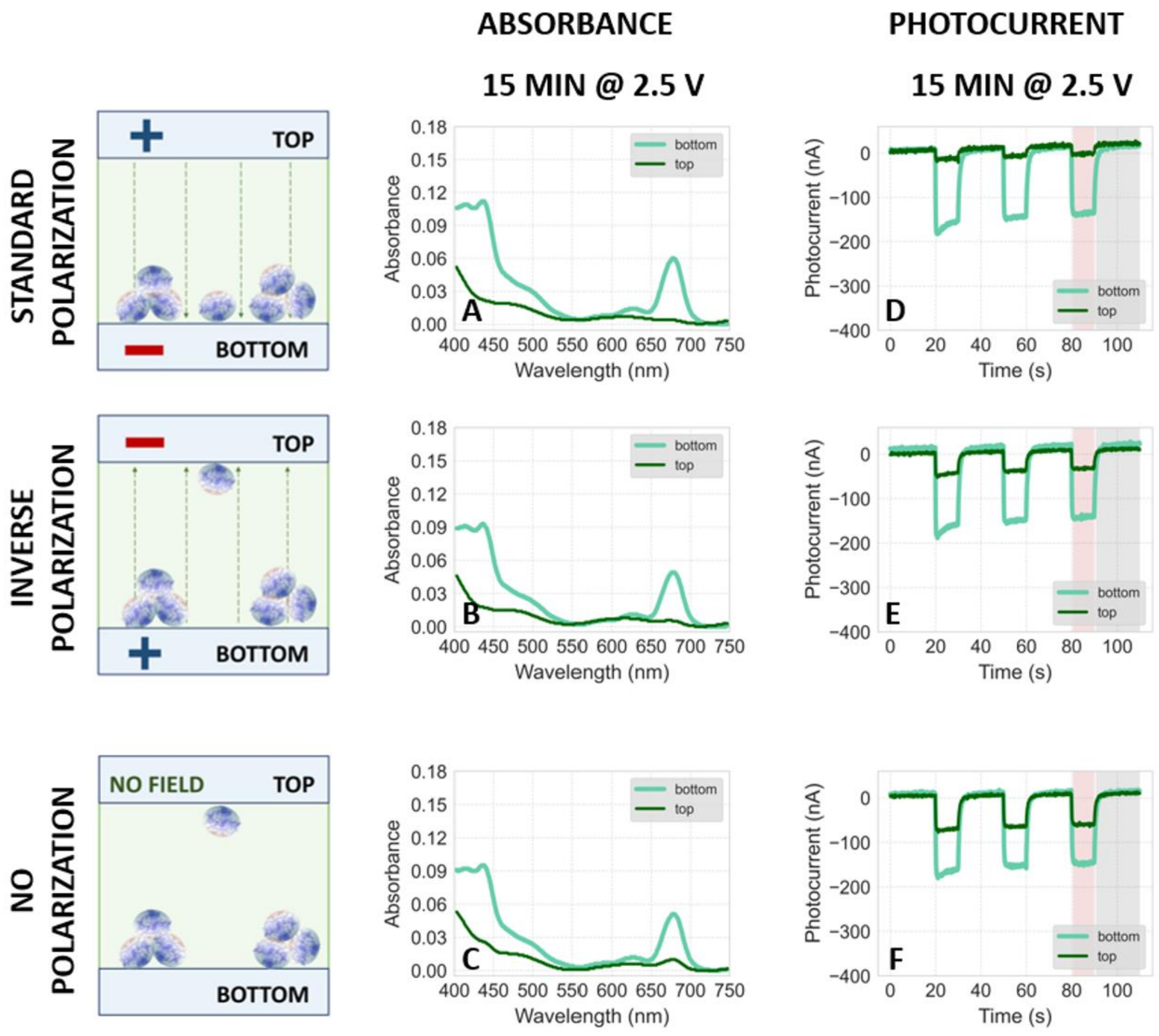

Figure S6. (A-E) Comparison of typical steady-state absorption spectra of PSI-FTO plates obtained by 15 minutes electrodeposition with standard (A), inverse (B) and no polarization (C) at **pH 5**. The light green curves represent the spectra of the bottom plates and the dark green ones represent the top plates. A voltage of 2.5 V was applied (panels A and B). In the case of no voltage applied (panel C), the setup – composed of two plates and a droplet of PSI suspension between them – was assembled for the same amount of time as those with the voltage applied. The schemes on the left-hand side show to which FTO plates most of the PSI particles migrate and are attached, after electrodeposition and evaporation (see Fig. 2), under the indicated polarization conditions. (D-F) Comparison of photochronoamperometric results obtained for PSI-FTO electrodes characterized spectroscopically in panels A-C, respectively. Note that the sample investigated at pH 5 was stored with glycerol (see Materials and methods).
